# High-dose rifampicin, moxifloxacin, and SQ109 for treating tuberculosis: a multi-arm, multi-stage randomised controlled trial

**DOI:** 10.1016/S1473-3099(16)30274-2

**Published:** 2017-01

**Authors:** Martin J Boeree, Norbert Heinrich, Rob Aarnoutse, Andreas H Diacon, Rodney Dawson, Sunita Rehal, Gibson S Kibiki, Gavin Churchyard, Ian Sanne, Nyanda E Ntinginya, Lilian T Minja, Robert D Hunt, Salome Charalambous, Madeleine Hanekom, Hadija H Semvua, Stellah G Mpagama, Christina Manyama, Bariki Mtafya, Klaus Reither, Robert S Wallis, Amour Venter, Kim Narunsky, Anka Mekota, Sonja Henne, Angela Colbers, Georgette Plemper van Balen, Stephen H Gillespie, Patrick P J Phillips, Michael Hoelscher, PanACEA consortium

**Affiliations:** a Department of Lung Diseases, Radboud University Medical Center, Nijmegen, Netherlands; b Department of Pharmacy, Radboud University Medical Center, Nijmegen, Netherlands; c Division of Infectious Diseases and Tropical Medicine, Medical Centre of the University of Munich, Munich, Germany; d German Center for Infection Research, Partner Site Munich, Germany; e Centre for Clinical Tuberculosis Research, Department of Science and Technology and National Research Foundation Centre of Excellence for Biomedical Tuberculosis Research, Faculty of Health Sciences, University of Stellenbosch, Tygerberg, South Africa; f MRC Centre for Tuberculosis Research, University of Stellenbosch, Tygerberg, South Africa; g Centre for Tuberculosis Research Innovation, University of Cape Town, Grote Schuur, South Africa; h Kilimanjaro Clinical Research Institute, Kilimanjaro Christian Medical Centre, Tumaini University, Moshi, Tanzania; i Kibong'oto National Tuberculosis Hospital, Tanzania; j National Institute for Medical Research, Mbeya Medical Research Centre, Mbeya, Tanzania; k Swiss Tropical and Public Health Institute, Basel, Switzerland; l University of Basel, Basel, Switzerland; m Ifakara Health Institute, Bagamoyo, Tanzania; n Division of Infection and Immunity, Centre for Clinical Microbiology, University College London, UK; o MRC Clinical Trials Unit at UCL, London, UK; p Medical School University of St Andrews, North Haugh, St Andrews, UK; q Aurum Institute, Johannesburg, South Africa; r School of Public Health, University of Witwatersrand, Johannesburg, South Africa; s Helen Joseph Hospital, Johannesburg, South Africa; t Department of Infectious Diseases, London School of Hygiene & Tropical Medicine, London, UK

## Abstract

**Background:**

Tuberculosis is the world's leading infectious disease killer. We aimed to identify shorter, safer drug regimens for the treatment of tuberculosis.

**Methods:**

We did a randomised controlled, open-label trial with a multi-arm, multi-stage design. The trial was done in seven sites in South Africa and Tanzania, including hospitals, health centres, and clinical trial centres. Patients with newly diagnosed, rifampicin-sensitive, previously untreated pulmonary tuberculosis were randomly assigned in a 1:1:1:1:2 ratio to receive (all orally) either 35 mg/kg rifampicin per day with 15–20 mg/kg ethambutol, 20 mg/kg rifampicin per day with 400 mg moxifloxacin, 20 mg/kg rifampicin per day with 300 mg SQ109, 10 mg/kg rifampicin per day with 300 mg SQ109, or a daily standard control regimen (10 mg/kg rifampicin, 5 mg/kg isoniazid, 25 mg/kg pyrazinamide, and 15–20 mg/kg ethambutol). Experimental treatments were given with oral 5 mg/kg isoniazid and 25 mg/kg pyrazinamide per day for 12 weeks, followed by 14 weeks of 5 mg/kg isoniazid and 10 mg/kg rifampicin per day. Because of the orange discoloration of body fluids with higher doses of rifampicin it was not possible to mask patients and clinicians to treatment allocation. The primary endpoint was time to culture conversion in liquid media within 12 weeks. Patients without evidence of rifampicin resistance on phenotypic test who took at least one dose of study treatment and had one positive culture on liquid or solid media before or within the first 2 weeks of treatment were included in the primary analysis (modified intention to treat). Time-to-event data were analysed using a Cox proportional-hazards regression model and adjusted for minimisation variables. The proportional hazard assumption was tested using Schoelfeld residuals, with threshold p<0·05 for non-proportionality. The trial is registered with ClinicalTrials.gov (NCT01785186).

**Findings:**

Between May 7, 2013, and March 25, 2014, we enrolled and randomly assigned 365 patients to different treatment arms (63 to rifampicin 35 mg/kg, isoniazid, pyrazinamide, and ethambutol; 59 to rifampicin 10 mg/kg, isoniazid, pyrazinamide, SQ109; 57 to rifampicin 20 mg/kg, isoniazid, pyrazinamide, and SQ109; 63 to rifampicin 10 mg/kg, isoniazid, pyrazinamide, and moxifloxacin; and 123 to the control arm). Recruitment was stopped early in the arms containing SQ109 since prespecified efficacy thresholds were not met at the planned interim analysis. Time to stable culture conversion in liquid media was faster in the 35 mg/kg rifampicin group than in the control group (median 48 days *vs* 62 days, adjusted hazard ratio 1·78; 95% CI 1·22–2·58, p=0·003), but not in other experimental arms. There was no difference in any of the groups in time to culture conversion on solid media. 11 patients had treatment failure or recurrent disease during post-treatment follow-up: one in the 35 mg/kg rifampicin arm and none in the moxifloxacin arm. 45 (12%) of 365 patients reported grade 3–5 adverse events, with similar proportions in each arm.

**Interpretation:**

A dose of 35 mg/kg rifampicin was safe, reduced the time to culture conversion in liquid media, and could be a promising component of future, shorter regimens. Our adaptive trial design was successfully implemented in a multi-centre, high tuberculosis burden setting, and could speed regimen development at reduced cost.

**Funding:**

The study was funded by the European and Developing Countries Clinical Trials partnership (EDCTP), the German Ministry for Education and Research (BmBF), and the Medical Research Council UK (MRC).

## Introduction

Tuberculosis is now the leading infectious disease killer worldwide. Treatment regimens last at least 6 months, so shorter, safer, and more effective regimens for drug-sensitive tuberculosis are needed as part of the global strategy to eliminate the disease.

Rifampicin is a key drug that, combined with pyrazinamide, reduced tuberculosis treatment from 18 to 6 months.^1^, ^2^ The standard dose of rifampicin (10 mg/kg) was chosen in the 1960s, primarily because of cost.^2^ However, results of several studies in mice^3^, ^4^, ^5^, ^6^ showed that higher doses can accelerate cure, and higher doses seemed to increase sputum culture conversion in clinical trials.^7^ In a dose ranging trial,^8^ 35 mg/kg showed increased efficacy and good tolerability when administered daily for 14 days. In another phase 2 study^9^ of 600 mg (10 mg/kg), 900 mg (15 mg/kg), and 1200 mg (20 mg/kg) rifampicin with standard concomitant treatment, patients showed good tolerability but no difference in efficacy in the three groups.

**Research in context**

**Evidence before this study**

We did literature searches in PubMed, applying the Medical Subject Heading (MeSH) terms “tuberculosis’ and “rifampicin AND dose”, “moxifloxacin”, or “SQ109”. Publications on pulmonary tuberculosis listed not later than April 30, 2016, in English, German, French, and Italian language were considered.

Pharmacokinetic studies indicate that standard dose (1 0mg/kg) of rifampicin often did not achieve effective plasma concentrations in patients. Enhanced efficacy of higher doses of rifampicin was reported in a number of animal studies. A systematic review on elevated doses of rifampicin published before 2008 identified 14 studies testing up to 1200 mg of rifampicin. Despite difficulties comparing efficacy outcomes across trials, there was an indication that higher doses were beneficial. Among 339 articles published after 2008, a single 14-day dose-ranging study of up to 35 mg/kg reported a supra-proportional increase in pharmacokinetic parameters and good tolerability, with a suggestion of enhanced early bactericidal activity. 43 publications on SQ109 were identified. A mouse study reported improved efficacy when SQ109 replaced ethambutol, but with a delayed onset of several weeks in reduction of lung and spleen colony-forming units. One 14-day phase 1 and one phase 2a study reported good tolerability, but absence of early bactericidal activity during the 14-day phase 2a study duration. A meta-analysis of moxifloxacin trials concluded that this drug added to the bactericidal activity of the regimen when it replaced ethambutol or isoniazid, but this was not enough to shorten treatment duration from 6 to 4 months.

**Added value of this study**

This study showed that 35 mg/kg rifampicin given over 12 weeks was safe and shortened the time to stable culture conversion from 62 to 48 days, showing the potential for an enhanced regimen. The other experimental arms, including various combinations of 10 mg/kg or 20 mg/kg of rifampicin, moxifloxacin, and SQ109, did not achieve significant improvements over the control arm. Taking all the data into consideration, this study supports that rifampicin given at 35 mg/kg is likely to improve treatment outcome. To our knowledge, this is the first time that a multi-arm adaptive trial design was successfully implemented in a multi-centre study in a high tuberculosis burden setting. This approach might accelerate tuberculosis regimen development at a reduced cost.

**Implications of all the available evidence**

Our study substantiated that an increase in rifampicin dose could improve the clearance of bacteria in patients with pulmonary tuberculosis without an increase in associated adverse events. The scale of the improvement shown in this study of 35 mg/kg rifampicin administered orally could translate to improved clinical outcomes. Smaller increases in rifampicin doses did not have this effect and suggest that future pivotal phase 3 studies should be done with at least 35 mg/kg. The combination of moxifloxacin and 20 mg/kg rifampicin had a modest effect on bacterial clearance. This combination could be improved by increasing the dose of moxifloxacin to overcome induction of its metabolism by rifampicin.

To address the challenge of choosing among the many potential drug combinations that should be assessed in phase 3 clinical trials, we adapted a multi-arm, multi-stage trial design^10^ that has been used successfully in oncology,^11^ and in which multiple regimens are compared with a common standard regimen. Recruitment to insufficiently efficacious regimens is discontinued if prespecified thresholds are not achieved to save time and resources, and to reduce the risk of exposing patients to an ineffective treatment. The objective of this approach is to generate data to select a regimen that might be eligible to progress to a pivotal phase 3 trial. We selected regimens based on literature and on previous studies within our consortium PanACEA (Pan African Consortium for the Evaluation of Antituberculosis Antibiotics). Regimens assessed in our trial also included 300 mg SQ109 (Sequella, Rockville, MD), a well-tolerated drug candidate based on the ethylene diamine pharmacopore.^12^, ^13^ Moxifloxacin was chosen to be assessed in combination with 20 mg/kg rifampicin. Moxifloxacin is a licensed antibiotic that leads to faster culture conversion when substituted for either isoniazid or ethambutol.^14^, ^15^, ^16^

We present the results of the PanACEA MAMS-TB trial, in which we aim to investigate four new potential regimens and establish a new pathway for tuberculosis drug regimen development.

## Methods

### Study design

We did a randomised, controlled, open-label, multi-arm, multi-stage study (MAMS) in three clinical trial sites in Tanzania and four trial sites in South Africa, including hospitals, health centres, and clinical trial units. The protocol was approved by independent ethics committees of the sponsor and the trial sites, and regulatory authorities of Tanzania and South Africa, and done according to Good Clinical Practice guidelines.^17^ The protocol is available in the appendix and panacea-tb.net.

### Patients

Eligible patients were aged 18 years or older, weighed 35–90 kg, had newly diagnosed, previously untreated pulmonary tuberculosis confirmed to be rifampicin sensitive by Xpert MTB/RIF, and positive smear microscopy of at least 1+ on the IUATLD/WHO scale. Patients with HIV were eligible if their CD4 count was greater than 200 cells per μL, and where local ethics committees agreed that antiretroviral treatment could be safely withheld until study week 12. Female patients were excluded if they were pregnant or breastfeeding. Patients were excluded if they received or required therapy expected to prolong the QT interval in electrocardiogram (ECG), or alter cytochrome P450 enzyme activity with potential effects on SQ109 metabolism (appendix p 7). Patients were recruited by invitation if diagnosed with tuberculosis in the public health system and included if they gave written informed consent

### Randomisation and masking

Patients were randomly assigned centrally using a web-based computerised algorithm, developed and maintained by the MRC Clinical Trials Unit. We used a random element of 80% for minimisation, stratified on study site, baseline bacterial load reported by Xpert MTB/RIF (high *vs* low; high is <16 cycle threshold), and HIV status. Eligible patients were assigned in a 1:1:1:1:2 ratio to one of four daily experimental regimens or a daily control regimen. Because of the orange discoloration of body fluids with higher doses of rifampicin it was not possible to mask patients and clinicians to treatment allocation. However, all laboratory assessments were done blind to treatment allocation and only the independent data monitoring committee (IDMC) and the trial statisticians saw data aggregated by treatment arm during the trial.

### Procedures

All drugs were oral, given once daily, 7 days per week. Control treatment consisted of standard dose rifampicin (10 mg/kg [range 8·1–11·8 mg/kg]), isoniazid (5 mg/kg [4·1–5·9 mg/kg]), pyrazinamide (25 mg/kg [21·6–31·6 mg/kg]), and ethambutol (15–20 mg/kg [14·9–21·7 mg/kg]) for 8 weeks, followed by 18 weeks of standard dose rifampicin and isoniazid. Experimental treatments consisted of rifampicin 35 mg/kg, plus standard dose isoniazid, pyrazinamide, and ethambutol (arm RIF_35_HZE); rifampicin 10 mg/kg, standard dose isoniazid and pyrazinamide, and SQ109 300 mg (arm RIFQHZ); rifampicin 20 mg/kg, standard dose isoniazid and pyrazinamide, and SQ109 300 mg (arm RIF_20_QHZ); or rifampicin 20 mg/kg, standard dose isoniazid and pyrazinamide, and moxifloxacin 400 mg (arm RIF_20_MHZ). Experimental treatment was given for 12 weeks, followed by 14 weeks of standard dose isoniazid and rifampicin. Pyridoxine (vitamin B6, 25 mg) was given in the morning to all patients throughout the trial. The control regimen was weight banded according to the South African tuberculosis treatment guidelines^18^ and implemented in both participating countries (appendix p 10). Higher doses of rifampicin were adapted to these weight bands (appendix). All drugs were self-administered except for days of clinic visits, where administration was directly observed. Participants were advised to take their drugs in the morning after a light breakfast and with a glass of water.

The primary study objective was to assess whether the experimental regimens, given for 12 weeks, resulted in shorter time to sputum culture conversion in liquid media compared with standard treatment. Patients were seen once per week up to week 12, and at weeks 14, 17, 22, and 26 after start of treatment. Sputum for smear and culture was taken 2 days before start of treatment, and at all visits. Samples were processed and cultured in liquid broth medium culture according to the mycobacteria growth indicator tube (Bactec MGIT960) system, on Löwenstein-Jensen (LJ) solid medium; and sensitivity to isoniazid, rifampicin, and ethambutol was assessed by liquid culture susceptibility testing (SIRE) at baseline and positive cultures after week 12, following the procedures described previously.^15^ Safety assessments included physical examination and vital signs. Liver function tests, lipase, electrolytes, glucose, and haematology were done at screening and treatment weeks 1, 2, 4, 6, 9, 12, and 14. Coagulation assessments were done at screening and at week 2, and ECGs at screening, baseline, and at week 1 and 2. The Friderica formula was used for heart rate correction of QT.^19^ All laboratory staff and ECG analysts were blinded to treatment allocation. Post-treatment follow-up was introduced during enrolment, following the IDMC review of the results of the first interim analysis. Follow-up was done at 3 months and 6 months after end of treatment by telephone, and patients were invited to attend the clinic for assessment if feeling unwell.

An electronic source documentation system (Clinical Ink, Winston Salem, NC), was used for clinical and laboratory data capture. All data were stored in a central study database to facilitate regular monitoring of data quality and completeness.

### Outcomes

The primary endpoint was time from treatment initiation to the first of two consecutive negative once-weekly sputum cultures without an intervening positive culture in liquid media, up to 12 weeks. Secondary endpoints were time to first negative culture in liquid and solid media, the proportion of patients converting to negative sputum culture in liquid and solid media at each time-point during treatment, rate of change in time to positivity in liquid culture and frequency of treatment failures or development of drug resistance in the different experimental arms, and safety. Modelling of rate of change in time to positivity in liquid culture is ongoing and will be reported more fully in a subsequent modelling paper.

Mycobacteriology endpoints were acquisition of resistance against rifampicin, pyrazinamide, isoniazid, ethambutol, and moxifloxacin during therapy. Minimum inhibitory concentrations and their change over treatment, and strain typing by genome sequencing that allows true relapse to be distinguished from reinfection are other endpoints that are being assessed and will be reported in a subsequent paper.

Adverse events were registered according to the Common Terminology Criteria for Adverse Events 4.03 (CTCAE).^20^ An adverse event was classified as serious if it led to death, permanent or significant disability, was a congenital anomaly or birth defect, was life threatening, or required hospital admission for management. Safety and efficacy results were reviewed by the IDMC at 6-monthly intervals during the trial. A trial steering committee with independent chair and majority supervised the conduct of the trial and made decisions following recommendations from the IDMC.

### Pharmacokinetics sub-study

20 patients allocated to each study arm, divided equally between the South African and Tanzanian sites, were enrolled into a pharmacokinetics sub-study where blood was taken at treatment week 4. A full description of the pharmacokinetic sub-study is provided in the appendix (pp 13–15).

### Statistical analysis

We assumed culture conversion of 85% by 12 weeks in the control arm^14^ and a 5% loss to follow-up, which meant that 124 patients in the control arm and 62 in the experimental arm would be adequate to detect a hazard ratio of 1·8 with 90% power and a two-sided type I error of 5% for each pairwise comparison (a total of 372 patients). We selected a hazard ratio (HR) of 1·8 as a criterion to indicate potential for treatment shortening, since this represented an increase over the 1·68 HR obtained in the moxifloxacin-containing phase 2 trial that led to the phase 3 REMoxTB trial.^14^, ^18^

Up to two interim IDMC analyses were planned after 28 and then 50 patients from the control arm had achieved stable culture conversion endpoint. Recruitment was to be terminated in experimental arms with HR less than 1·09 (first interim) or less than 1·23 (second interim). The probability of continuing an efficacious arm at each interim analysis—the stage-wise power—was 0·95, and the probability of not dropping an inefficacious arm—the stage-wise type I error—was 0·4 (first interim) and 0·2 (second interim).

Patients without evidence of rifampicin resistance on phenotypic test who took at least one dose of study treatment and had one positive culture on liquid or solid media before or within the first 2 weeks of treatment were included in the primary analysis population (a modified intention-to-treat [ITT] population). In secondary analyses, the primary endpoint was also analysed on an ITT population (all randomly assigned patients) and a per-protocol population (PP, the modified ITT population, excluding randomly assigned patients that did not meet the eligibility criteria and patients that missed 21 or more doses of their allocated treatment in the first 12 weeks). The modified ITT population was not included in the original trial protocol, but was included as the primary analysis population in the statistical analysis plan before any interim analyses to allow comparison with other tuberculosis trials. Including patients without culture-confirmed disease (ITT) or excluding patients based on post-randomisation data (PP) can introduce bias, and therefore the modified ITT population was preferred. Time-to-event data were analysed using a Cox proportional-hazards regression model and adjusted for minimisation variables (HIV status, Xpert MTB/RIF cycle threshold, centre) and for the baseline liquid culture bacterial load measurement, time to positivity (mean of pre-treatment cultures) for analyses of liquid culture data. Log-rank statistics were also calculated for time-to-event data, both unstratified and stratified by minimisation variables. The proportional-hazards assumption was tested using Schoelfeld residuals, with p<0·05 evidence for non-proportionality. Details of secondary outcomes and sensitivity analyses are described in the appendix. Post-hoc analyses for time to stable culture conversion to 8 weeks on liquid and solid media that were not prespecified in the statistical analysis plan were done for comparability with previous phase 2 studies.

To assess safety, the proportion of adverse events and the proportion of adverse events assessed by the investigators as probably related or related to treatment was presented by group. Data were analysed using Stata version 13.1 (StataCorp, College Station, Texas).

### Role of the funding source

The funders of the study had no role in study design, data collection, data analysis, data interpretation, or writing of the report. The corresponding author had full access to all the data in the study and had final responsibility for the decision to submit for publication. Sequella, Inc provided SQ109. Sequella had no role in study design, data collection, data analysis, and data interpretation; they reviewed the paper and made comments.

## Results

**Figure 1:**
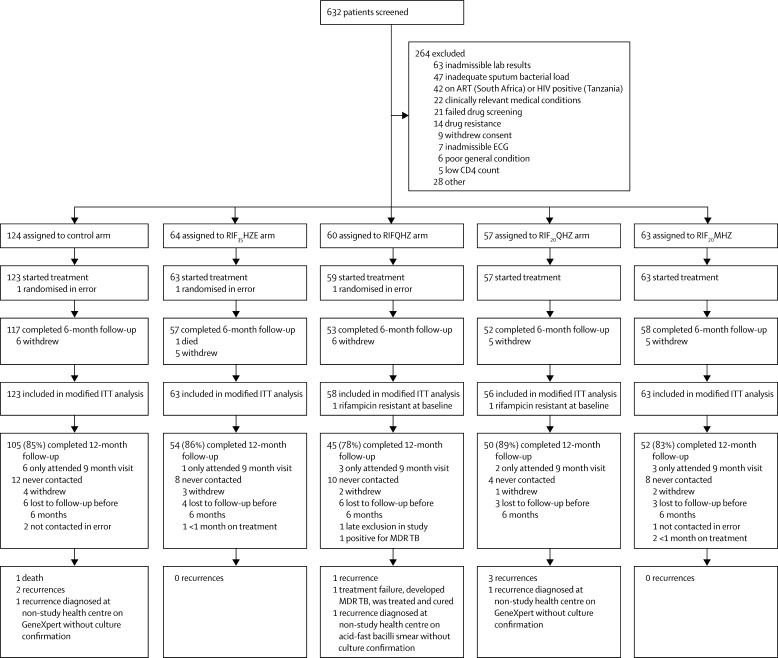
Trial profile Recruitment to RIFQHZ arm and RIF_20_QHZ arm was stopped early following the first interim analysed. Three patients who were randomised in error were not started on treatment and not retained in follow-up. RIF_35_HZE=rifampicin 35 mg/kg, isoniazid, pyrazinamide, ethambutol. RIFQHZ=rifampicin 10 mg/kg, isoniazid, pyrazinamide, SQ109 300 mg. RIF_20_QHZ=rifampicin 20 mg/kg, isoniazid, pyrazinamide, SQ109 300 mg. RIF_20_MHZ=rifampicin 20 mg/kg, isoniazid, pyrazinamide, moxifloxacin 400 mg. ITT=intention-to-treat. MDR TB=multi-drug resistant tuberculosis. Doses of concomitant drugs are detailed in Procedures.

Between May 7, 2013, and March 25, 2014, 632 patients were screened and 365 were enrolled in Tanzania (51 in Moshi, 52 in Mbeya, and 53 in Bagamoyo) and South Africa (88 in Cape Town, 48 in Stellenbosch, 30 in Johannesburg, and 43 in Tembisa). Three additional patients were randomised in error and were not started on treatment and not continued in follow-up (figure 1). Recruitment to the RIFQHZ and RIF_20_QHZ arms was terminated following the recommendation from the IDMC after review of results from the first scheduled interim analysis; however, enrolled patients continued on allocated treatment. Recruitment to all other arms continued until the target sample sizes in each arm had been reached. Recruitment was completed before the scheduled second interim analysis, which was therefore not done.

Baseline characteristics were similar between arms (table 1). Two patients were rifampicin resistant on phenotypic tests and were excluded from the modified ITT analysis population.

**Figure 2:**
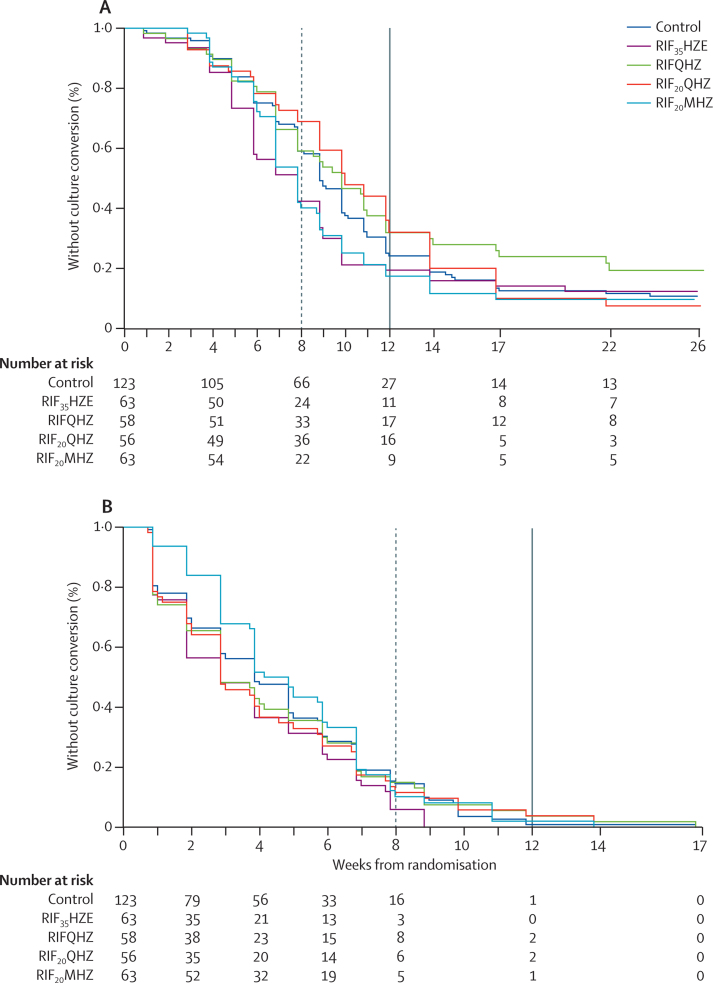
Kaplan-Meier curve for time to culture conversion (A) Time to culture conversion in liquid MGIT media. (B) Time to culture conversion on solid Löwenstein-Jensen media. MGIT=mycobacteria growth indicator tube. RIF_35_HZE=rifampicin 35 mg/kg, isoniazid, pyrazinamide, ethambutol. RIFQHZ=rifampicin 10 mg/kg, isoniazid, pyrazinamide, SQ109 300 mg. RIF_20_QHZ=rifampicin 20 mg/kg, isoniazid, pyrazinamide, SQ109 300 mg. RIF_20_MHZ=rifampicin 20 mg/kg, isoniazid, pyrazinamide, moxifloxacin 400 mg. Doses of concomitant drugs are detailed in Procedures. Dashed vertical line refers to the week 8 time-point (cutoff in post-hoc analysis). Solid vertical line refers to the week 12 time-point (cutoff in primary analysis).

Patients achieved faster stable culture conversion in MGIT on the RIF_35_HZE arm than on the control arm, with a median time to stable culture conversion of 48 days compared with 62 days (adjusted HR 1·78, 95% CI 1·22–2·58; p=0·003, table 2, figure 2). HR was smaller in the unadjusted analysis (HR 1·46, 95% CI 1·02–2·11; p=0·04). None of the other arms reported significantly faster culture conversion than control, with the largest effect seen in the RIF_20_MHZ arm (adjusted HR 1·42, 95% CI 0·98–2·05; p=0·07). The cumulative proportion of patients achieving the primary endpoint of culture conversion in liquid media at week 12 (using the Kaplan-Meier estimator) was 70·1% (control), 79·9% (RIF_35_HZE), 65·2% (RIFQHZ), 58·6% (RIF_20_QHZ), and 78·7% (RIF_20_MHZ).

Time to culture conversion on solid media did not differ significantly between any of the arms and the control arm. In the post-hoc analysis for comparability with previous tuberculosis phase 2 trials^14^, ^21^ at 8 weeks, HR for the primary endpoint on liquid culture was higher for RIF_35_HZE (adjusted HR 2·06, 95% CI 1·26–3·38; p=0·004) and for RIF_20_MHZ (1·67, 1·01–2·67; p=0·05, table 3). None of the arms reported significantly faster time to culture conversion than the control arm on solid media when data were censored at 8 weeks. To understand the differences in results between solid and liquid media, we did a further post-hoc analysis of time to culture conversion by excluding patients without a positive culture on solid media before or within the 2 weeks of randomisation; this analysis gave HRs that were marginally closer than those in liquid media, but differences between solid and liquid media remained (table 3). Results of further sensitivity analysis for the primary outcome and the secondary outcomes time to first negative culture in liquid and solid media and proportion of patients converting to negative sputum culture in liquid and solid media at each timepoint during treatment are presented in the appendix (pp 16–18).

One patient with baseline isoniazid mono-resistance treated with RIFQHZ acquired rifampicin and pyrazinamide resistance and was classified as treatment failure at week 17. The patient received appropriate treatment for multi-drug resistant tuberculosis and was reported cured after follow-up. Acquired drug resistance was not seen in any other patients. Seven patients were diagnosed with recurrent disease at 12 months after randomisation, and three more were identified by non-study health facilities without culture confirmation (one by smear and two by Xpert MTB/RIF; figure 1). Of these 11 unfavourable outcomes, only one occurred on the RIF_35_HZE arm and none on the RIF_20_MHZ arm, and all had longer time to conversion to negative culture in liquid media (median 97 days *vs* 62 days in those without failure or recurrence, p=0·013), but not on solid media (median 27 days *vs* 27 days, p=0·157).

45 patients reported at least one grade 3, 4, or 5 adverse event with similar proportions in each arm (table 4). There was one death during the trial, a patient on the RIF_35_HZE arm who died 13 weeks after experimental treatment was completed, near the end of the continuation phase, and who complained of sudden onset chest pain hours before death. This death was unlikely to be related to the higher dose of rifampicin. One patient in the control arm completed treatment successfully but later relapsed and died from underlying pneumoconiosis, related to work as a miner in Tanzania. Eight patients with hepatic adverse events fulfilled the criteria for treatment interruption (table 4). Investigators opted for treatment interruption in two additional cases of grade 2 elevated transaminases in arm RIF_35_HZE, in whom the protocol toxicity criteria for interruption were not fulfilled. Five patients were restarted on study treatment, which was tolerated by three. No QTcF interval of more than 480 ms was recorded during the study. Further details of adverse events are in the appendix (p 12).

Increasing the rifampicin dose from 10 to 35 mg/kg resulted in a more than proportional increase in the geometric mean area under the curve (AUC)_0–24h_ and maximum concentration (C_max_) of rifampicin [appendix, pp 12–15, 21]. No significant differences in isoniazid, pyrazinamide, or ethambutol exposure were found between standard and higher dose rifampicin arms, indicating that higher doses of rifampicin do not affect the exposures to these drugs. Tanzanians had a lower rifampicin AUC_0–24h_ than South Africans in the RIF_35_HZE arm (geometric means 145 and 206 h × mg/L, 11 *vs* 9 patients, p=0·001). Pyrazinamide AUC_0–24h_ values were also lower in Tanzanians than South Africans (317 and 360 h × mg/L, 94 patients, p=0·02). Plasma concentrations of SQ109 in the study are not yet available, a pharmacokinetic-pharmacodynamic analysis is ongoing, and will be reported separately.

## Discussion

Our study shows that a regimen including rifampicin 35 mg/kg resulted in significantly faster liquid culture conversion by 12 weeks. Previous tuberculosis trials have used culture conversion at 8 weeks, and the 8-week adjusted HR of 2·06 in our trial is the highest reported for any tuberculosis regimen; HRs from other trials include 1·68 for pretomanid,^21^ 1·52 for gatifloxacin,^14^ and 1·68 for moxifloxacin.^14^ Only a study^22^ of high dose rifapentine reported similar activity in terms of increasing the proportion of patients with sputum culture conversion at 8 weeks. Culture conversion rates in our study at weeks 12 and 26 were lower than in comparable trials. One patient had treatment failure with persistent positive cultures, but the others did not achieve the endpoint because of the stringent definition used, which requires two negative cultures from successive visits, and is susceptible to missing culture results by contamination or missing sample.

None of the other arms in our study resulted in faster culture conversion in liquid or on solid culture compared with control. Although RIF_20_MHZ was the best of 20 mg/kg rifampicin regimens, it was not better than moxifloxacin containing regimens with standard dose rifampicin reported previously.^14^, ^15^ This could be due to relatively low exposure to moxifloxacin in our cohort, considering that moxifloxacin is metabolised by the phase 2 metabolising enzymes uridine diphosphate glucuronosyltransferase and sulphotransferase. Rifampicin is known to induce such phase 2 metabolic enzymes and it has been shown that rifampicin decreases the AUC of moxifloxacin by approximately 30%.^23^ The results in the RIF_20_MHZ arm suggest that 20 mg/kg rifampicin does not result in enhanced efficacy. SQ109 did not reach the prespecified HR in the dose and combinations used. This drug is metabolised by cytochrome P450 iso-enzymes CYP2D6 and CYP2C19, and at least CYP2C19 can be induced by rifampicin. We have previously shown that rifampicin decreases exposure to SQ109 administered in a 150 mg daily dose, but a higher daily dose of 300 mg SQ109, as used in our study, outweighed this inductive effect of rifampicin.^13^

Clinical outcome was assessed at 12 months post randomisation (6 months after completion of treatment) and recurrence rates were similar between regimens.

Faster conversion in the RIF_35_HZE arm was only seen with liquid but not with solid culture, unlike a large trial assessing moxifloxacin regimens where the culture conversion results were the same on liquid and solid media.^15^ Rifampicin is considered to be effective not only on actively replicating but also on persisting, non-replicating bacterial phenotypes that are drug tolerant.^24^ Incomplete killing of such bacilli is one of the proposed reasons for relapse after treatment completion^25^ as is poor penetration to the site of infection.^26^ Higher doses of rifampicin seem to more effectively kill this subpopulation in experimental models^4^, ^24^ and in human beings.^27^, ^28^ Since liquid media, unlike solid media, detect such bacilli,^29^ we believe that the higher efficacy of RIF_35_HZE seen in liquid media represents increased activity of this regimen on this bacterial phenotype. The relevance of the liquid media endpoint is further supported by the fact that patients with treatment failure or recurrent disease had later culture conversion on liquid media, but not on solid media (although numbers were small).

Increasing the dose of rifampicin from 10 to 35 mg/kg resulted in a more than proportional increase in exposure to rifampicin, which is in agreement with previous data^30^ and with more recent pharmacokinetic results from our rifampicin dose ranging trial.^8^ The response associated with higher doses and exposures to rifampicin is also in agreement with in-vitro, infected macrophage, and in-vivo murine data that showed an increased bactericidal effect and shortening of treatment duration once the steep part of the rifampicin dose–exposure-response curve is reached.^3^, ^6^ In this respect, the 35 mg/kg rifampicin regimen is promising but might not yet be optimal. Our ongoing phase 2a dose escalating study indicates that a higher dose of 40 mg/kg is also safe and well tolerated.^8^, ^9^ The rifampicin dose might yet be increased safely upon the human dose–exposure-response curve, which could aid treatment shortening and reduce the risk of rifampicin resistance for individuals with lower drug exposures.^31^ Our data bring into question the utility of testing regimens with only 20 mg/kg rifampicin,^9^ suggesting that they are not likely to provide significant benefit or treatment shortening. A trial^32^ from Vietnam in tuberculosis meningitis did not find a reduction in mortality with a regimen containing 15 mg/kg oral rifampicin.^32^ A higher dose and exposure to rifampicin would also lessen the relevance of the wide inter-individual variability in exposure to rifampicin as observed in this study. Higher AUCs were observed in South Africa than in Tanzania in the 35 mg/kg arm, possibly associated with inter-ethnic differences in genetic polymorphisms of genes encoding for drug transporters or enzymes involved in the metabolism of rifampicin.^33^

The advantage of the concept of high dose rifampicin is that the drug is widely available at low cost and implementation could take place broadly and quickly. A larger pill burden is a potential disadvantage, as available rifampicin formulations are not adapted to the higher doses. After defining the optimal dose of rifampicin new formulations would need to be developed in fixed dose combinations with companion drugs.

Experimental arms had similar safety profiles to the control arm. Treatment interruptions due to hepatic events were few. Although more interruptions occurred in the RIF_35_HZE arm, in two instances the severity was less than specified by the protocol, and since clinicians were not blinded to treatment allocation this might have influenced the decision to stop treatment.

Our study was, to our knowledge, the first multi-arm, multi-stage trial in infectious diseases, and showed that this design is feasible in a multicentre African setting, and can be used to assess many novel combinations. We identified a regimen that achieved our target and has promise to progress to phase 3, while discontinuing two regimens that did not show sufficient efficacy, thereby saving on resources.

This study has some limitations. It was not powered to be able to differentiate between the experimental regimens, only between individual arms and the control regimen. The sample size to achieve such a power would be inappropriately large. Furthermore, it was not powered for assessment of relapse, which is the definitive endpoint of a pivotal trial but for time to culture conversion, which is an intermediate endpoint. Our newly developed phase 2c STEP trial design will address this issue in future trials,^34^ and will improve confidence in a decision to move an arm into phase 3 based on a relapse endpoint.

Also, our objective was to identify regimens for future phase 3 assessment and thus our study does not provide information on the activity of individual experimental components. The study was only done in Africa and the HIV co-infected population was small. Therefore, future phase 3 trials should include sites from outside Africa and recruit representative proportions of HIV co-infected patients on antiretroviral treatment.

Another limitation is that because of the time required from sputum collection to determine negative cultures (which was necessary for our primary endpoint) and due to faster than expected recruitment, the interim analysis occurred late in the recruitment period. As a result, the multi-arm, multi-stage design resulted in a reduction in the final sample size of only 2%. A real-time biomarker such as the molecular bacterial load assay could overcome this problem by replacing culture allowing the endpoint results to be available rapidly.^35^ Nevertheless, this was a crucial opportunity to assess the feasibility of this novel design in the context of an African multicentre infectious diseases clinical trial. In the future, larger trials with more arms, and higher patient numbers per arm will achieve more substantial cost savings through a multi-arm, multi-stage design. These would also produce smaller confidence intervals, allowing a more precise estimate of the true HR. The collection of relapse data piloted in this study has been incorporated into our newly developed phase 2c STEP trial design.^34^ Such developments will provide the data to support the decision to move an arm into phase 3.

We have shown that 35 mg/kg rifampicin is safe and reduces time to culture conversion in liquid media. Importantly, the multi-arm, multi-stage concept has been shown to be feasible in a multicentre tuberculosis high burden setting, generating evidence to make a decision for whether or not to proceed to phase 3, and could greatly speed regimen development at reduced cost. In this phase 3 trial, a regimen with high dose rifampicin can be tested for safety and tolerability, potential to shorten duration of treatment, and ability to prevent the emergence of resistance to rifampicin.

## Figures and Tables

**Table 1:** Baseline characteristics by arm

		**Control**	**RIF**_35_**HZE**	**RIFQHZ**	**RIF**_20_**QHZ**	**RIF**_20_**MHZ**	**Total**
Number randomised*		123	63	59	57	63	365
Age (years)		34 (26–41)	33 (23–40)	32 (25–40)	34 (27–41)	31 (24–38)	33 (26–40)
Male		94 (76%)	42 (67%)	38 (64%)	45 (79%)	39 (62%)	258 (71%)
Weight (Kg)		54 (49–59)	52 (47–58)	53 (47–57)	53 (49–56)	52 (48–61)	53 (49–58)
HIV positive		9 (7%)	4 (6%)	5 (8%)	3 (5%)	3 (5%)	24 (7%)
Ethnicity							
	Black	101 (82%)	51 (81%)	50 (85%)	50 (88%)	48 (76%)	300 (82%)
	White	0 (0%)	1 (2%)	0 (0%)	1 (2%)	0 (0%)	2 (1%)
	Mixed	19 (15%)	11 (17%)	9 (15%)	6 (11%)	15 (24%)	60 (16%)
	Other	3 (2%)	0 (0%)	0 (0%)	0 (0%)	0 (0%)	3 (1%)
Xpert MTB/RIF cycle threshold		16 (14–19)	17 (14–20)	17 (14–19)	16 (14–18)	16 (14–19)	16 (14–19)
Phenotypic resistance to rifampicin							
	Resistant	0 (0%)	0 (0%)	1 (2%)	1 (2%)	0 (0%)	2 (1%)
	Sensitive	112 (91%)	59 (94%)	54 (92%)	53 (93%)	56 (89%)	334 (92%)
	Missing	11 (9%)	4 (6%)	4 (7%)	3 (5%)	7 (11%)	29 (8%)
Phenotypic resistance to isoniazid							
	Resistant	3 (2%)	0 (0%)	3 (5%)	1 (2%)	1 (2%)	8 (2%)
	Sensitive	109 (89%)	59 (94%)	52 (88%)	53 (93%)	55 (87%)	328 (90%)
	Missing	11 (9%)	4 (6%)	4 (7%)	3 (5%)	7 (11%)	29 (8%)
Phenotypic resistance to moxifloxacin							
	Resistant	0 (0%)	0 (0%)	0 (0%)	0 (0%)	0 (0%)	0 (0%)
	Sensitive	115 (93%)	59 (94%)	55 (93%)	54 (95%)	56 (89%)	339 (93%)
	Missing	8 (7%)	4 (6%)	4 (7%)	3 (5%)	7 (11%)	26 (7%)
Phenotypic resistance to ethambutol							
	Resistant	0 (0%)	0 (0%)	0 (0%)	1 (2%)	0 (0%)	1 (0%)
	Sensitive	112 (91%)	59 (94%)	55 (93%)	53 (93%)	56 (89%)	335 (92%)
	Missing	11 (9%)	4 (6%)	4 (7%)	3 (5%)	7 (11%)	29 (8%)
Phenotypic resistance to pyrazinamide							
	Resistant	4 (3%)	2 (3%)	2 (3%)	0 (0%)	4 (6%)	12 (3%)
	Sensitive	110 (89%)	57 (90%)	53 (90%)	55 (96%)	53 (84%)	328 (90%)
	Missing	9 (7%)	4 (6%)	4 (7%)	2 (4%)	6 (10%)	25 (7%)

* An additional three patients were randomised in error and did not start treatment or remain in follow-up and are therefore not included in this table. RIF_35_HZE=rifampicin 35 mg/kg, isoniazid, pyrazinamide, ethambutol. RIFQHZ =rifampicin 10 mg/kg, isoniazid, pyrazinamide, SQ109 300 mg. RIF_20_QHZ=rifampicin 20 mg/kg, isoniazid, pyrazinamide, SQ109 300 mg. RIF_20_MHZ=rifampicin 20 mg/kg, isoniazid, pyrazinamide, moxifloxacin 400mg. Data are median (IQR) or n (%) unless otherwise specified. Doses of concomitant drugs are detailed in Procedures.

**Table 2:** Summary of analyses of time to culture conversion in MGIT culture (primary) and on solid LJ culture (secondary) to 12 weeks

		**Control**	**RIF**_35_**HZE**	**RIFQHZ**	**RIF**_20_**QHZ**	**RIF**_20_**MHZ**	**Total**
Total in analysis (mITT)		123	63	58	56	63	363
Number of culture conversions during 26-week follow-up (MGIT culture)		101 (82%)	51 (81%)	44 (76%)	48 (86%)	52 (83%)	296 (82%)
Number of culture conversions during 26-week follow-up (solid culture)		117 (95%)	59 (94%)	59 (97%)	54 (96%)	59 (94%)	345 (95%)
Primary analysis to 12 weeks (MGIT culture)							
	Cumulative probability of culture conversion by 12 weeks	70·1%	79·9%	65·2%	58·6%	78·7%	..
	Median time to culture conversion (IQR)	62 (41–83)	48 (34–69)	63 (48–83)	66 (41–83)	55 (41–69)	..
	Adjusted hazard ratio (95%)*	..	1·78 (1·22–2·58) p=0·003	0·85 (0·57–1·27) p=0·42	0·76 (0·50–1·17) p=0·21	1·42 (0·98–2·05) p=0·07	..
	Hazard ratio (95%), unadjusted	..	1·46 (1·02–2·11) p=0·04	0·90 (0·60–1·34) p=0·60	0·76 (0·50–1·16) p=0·21	1·34 (0·93–1·93) p=0·12	..
Solid LJ culture to 12 weeks (secondary)							
	Cumulative probability of culture conversion by 12 weeks	97·3%	100·0%	94·4%	94·2%	98·0%	..
	Median time to culture conversion (IQR)	27 (13–48)	20 (7–41)	20 (7–48)	20 (11–44)	29 (20–48)	..
	Adjusted hazard ratio (95% CI)*	..	1·23 (0·89–1·69) p=0·21	0·91 (0·66–1·27) p=0·58	0·98 (0·70–1·38) p=0·93	0·77 (0·56–1·06) p=0·11	..
	Unadjusted hazard ratio (95% CI)	..	1·28 (0·93–1·75) p=0·13	1·02 (0·73–1·41) p=0·92	1·06 (0·76–1·47) p=0·74	0·90 (0·65–1·23) p=0·50	..

LJ=Löwenstein-Jensen. MGIT=mycobacteria growth indicator tube. mITT=modified intention to treat. RIF_35_HZE=rifampicin 35 mg/kg, isoniazid, pyrazinamide, ethambutol. RIFQHZ=rifampicin 10 mg/kg, isoniazid, pyrazinamide, SQ109 300 mg. RIF_20_QHZ=rifampicin 20 mg/kg, isoniazid, pyrazinamide, SQ109 300 mg. RIF_20_MHZ=rifampicin 20 mg/kg, isoniazid, pyrazinamide, moxifloxacin 400 mg. Doses of concomitant drugs are detailed in Procedures.

* Analysis adjusted for HIV status, GeneXpert cycle threshold (<16, ≥16), and site. MGIT analyses also adjusted for baseline time to positivity.

**Table 3:** Summary of analyses of time to culture conversion in MGIT and on LJ culture to 8 weeks (post hoc), and on LJ culture excluding patients without positive LJ at baseline (post hoc)

	**Control**	**RIF**_35_**HZE**	**RIFQHZ**	**RIF**_20_**QHZ**	**RIF**_20_**MHZ**
**MGIT culture censored at 8 weeks (post hoc)**					
Cumulative probability of culture conversion by 8 weeks	32%	49%	34·5%	27·8%	46·2%
Adjusted hazard ratio (95% CI)*	..	2·06 (1·26–3·38) p=0·004	1·04 (0·59–1·81) p=0·90	0·91 (0·49–1·67) p=0·76	1·67 (1·01–2·67) p=0·05
Unadjusted hazard ratio (95% CI)	..	1·73 (1·07–2·82) p=0·03	1·07 (0·62–1·86) p=0·81	0·87 (0·48–1·58) p=0·64	1·47 (0·90–2·40) p=0·13
**Solid LJ culture censored at 8 weeks (post hoc)**					
Cumulative probability of culture conversion by 8 weeks	80·9%	88·0%	83·9%	82·6%	82·7%
Adjusted hazard ratio (95% CI)*	..	1·17 (0·83–1·64)	1·00 (0·70–1·42)	1·06 (0·74–1·52)	0·76 (0·54–1·07)
Adjusted log-rank test*	..	p=0·38	p=1·00	p=0·75	p=0·12
Unadjusted hazard ratio (95% CI)	..	1·24 (0·88–1·73)	1·09 (0·77–1·55)	1·12 (0·79–1·60)	0·88 (0·63–1·24)
Unadjusted log-rank test	..	p=0·22	p=0·62	p=0·53	p=0·48
**Solid LJ culture censored at 12 weeks excluding without a positive culture on LJ solid media before or within the 2 weeks of randomisation (post hoc)**					
Number in analysis (total=297)	101	46	45	47	58
Cumulative probability of culture conversion by 8 weeks	96·7%	100·0%	92·8%	93·3%	97·8%
Adjusted hazard ratio (95% CI)*	..	1·37 (0·95–1·99)	0·84 (0·58–1·23)	1·00 (0·69–1·45)	0·88 (0·62–1·24)
Adjusted log-rank test*	..	p=0·19	p=0·78	p=0·62	p=0·37
Unadjusted hazard ratio (95% CI)	..	1·37 (0·95–1·98)	0·92 (0·64–1·34)	1·05 (0·73–1·51)	0·95 (0·67–1·33)
Log-rank test, unadjusted	..	p=0·07	p=0·65	p=0·76	p=0·73

LJ=Löwenstein-Jensen. MGIT=mycobacteria growth indicator tube. RIF_35_HZE=rifampicin 35 mg/kg, isoniazid, pyrazinamide, ethambutol. RIFQHZ=rifampicin 10 mg/kg, isoniazid, pyrazinamide, SQ109 300 mg. RIF_20_QHZ=rifampicin 20 mg/kg, isoniazid, pyrazinamide, SQ109 300 mg. RIF_20_MHZ=rifampicin 20 mg/kg, isoniazid, pyrazinamide, moxifloxacin 400 mg. Doses of concomitant drugs are detailed in Procedures.

* Analysis adjusted for HIV status, GeneXpert cycle threshold (<16, ≥16), and site. MGIT analyses also adjusted for baseline time to positivity.

**Table 4:** Summary of adverse events

	**Control**	**RIF**_35_**HZE**	**RIFQHZ**	**RIF**_20_**QHZ**	**RIF**_20_**MHZ**	**Total**
Total in safety analysis	123	63	59	57	63	365
Patients with at least one AE	92 (75%)	53 (84%)	49 (83%)	42 (74%)	49 (78%)	285 (78%)
Patients with at least one grade 3, 4, or 5 AE	13 (11%)	9 (14%)	7 (12%)	7 (12%)	9 (14%)	45 (12%)
Patients with at least one grade 3, 4, or 5 AE considered probably related or related	1 (1%)	3 (5%)	0	0	4 (6%)	8 (2%)
Patients with at least one serious AE	6 (5%)	4 (6%)	4 (7%)	5 (9%)	4 (6%)	23 (6%)
Deaths	0	1	0	0	0	1
Total number of patients with treatment changed due to hepatic AE	2 (2%)	5 (8%)	0 (0%)	3 (5%)	0	10 (3%)
Number of patients with treatment changed due to hepatic AE—symptomatic or meeting protocol criteria*	2 (2%)	3 (5%)	0 (0%)	3 (5%)	0	8 (2%)
Treatment changed due to hepatic AE—not fulfilling protocol criteria and not being symptomatic*	0	2 (3%)	0	0	0	(1%)

AE=adverse event. AST=aspartate aminotransferase. ALT=alanine transaminase. RIF_35_HZE=rifampicin 35 mg/kg, isoniazid, pyrazinamide, ethambutol. RIFQHZ=rifampicin 10 mg/kg, isoniazid, pyrazinamide, SQ109 300 mg. RIF_20_QHZ=rifampicin 20 mg/kg, isoniazid, pyrazinamide, SQ109 300 mg. RIF_20_MHZ=rifampicin 20 mg/kg, isoniazid, pyrazinamide, moxifloxacin 400 mg. Doses of concomitant drugs are detailed in Procedures.

* Protocol criteria for treatment interruption due to hepatic AE: elevation of AST or ALT more than three times, but less than five times the upper limit of normal, with associated symptoms or elevation of AST or ALT more than five times the upper limit of normal irrespective of the presence of symptoms.
