# Supplementary material for: High-dose rifampicin, moxifloxacin, and SQ109 for treating tuberculosis: a multi-arm, multi-stage randomised controlled trial
Source: Lancet Infect Dis. 2017 Jan;17(1):39–49. doi: 10.1016/S1473-3099(16)30274-2 (PMC5159618; doi:10.1016/S1473-3099(16)30274-2)

THE LANCET Infectious Diseases

Supplementary webappendix

This webappendix formed part of the original submission and has been peer reviewed. We post it as supplied by the authors.

Supplement to: Boeree MJ, Heinrich N, Aarnoutse R, et al, on behalf of the PanACEA consortium. High-dose rifampicin, moxifloxacin, and SQ109 for treating tuberculosis: a multi-arm, multi-stage randomised controlled trial. *Lancet Infect Dis* 2016; published online Oct 26. http://dx.doi.org/10.1016/S1473-3099(16)30274-2.

PanACEA MAMS-TB Online Supplement

Contents

The PanACEA Consortium 2

Supplementary methods: 4

Data management 4

Definition of primary endpoint 4

Sensitivity analyses and analysis of secondary endpoints 4

Pharmacokinetic sub-study 5

Supplementary Tables and Figures 7

Table S1: Inclusion and Exclusion criteria 7

Table S2: Study drug and tablet scheme for intensive/experimental and continuation phase 10

Table S3. Summary of grade 3 or above adverse events by MedDRA system organ class. 12

Table S4. Pharmacokinetics of TB drugs recorded after 4 weeks of treatment (steady-state) 13

Table S5. Summary of analyses of time to culture conversion under different definitions. 16

Table S6. Summary of primary analyses of time to culture conversion in ITT and PP populations. . 18

Table S7. Proportion of patients converting to negative sputum culture in liquid and solid media to 12 weeks at each time point during treatment. 19

Figure S1: Dose per body weight of all drugs dosed according to weight. 20

Figure S2. Pharmacokinetics of rifampicin 21

The PanACEA Consortium

The Pan African Consortium for the Evaluation of Anti-tuberculosis Antibiotics (PanACEA) comprises of the following individuals and institutions: Medical Centre of the University of Munich, Munich, Germany (Anna Maria Mekota, Norbert Heinrich, Andrea Rachow, Elmar Saathoff, Michael Hoelscher); University of St. Andrews, St Andrews, United Kingdom (Stephen Gillespie); Radboud University Nijmegen Medical Center, Nijmegen, the Netherlands (Georgette Plemper van Balen, Marloes Weijers, Angela Colbers, Rob Aarnoutse, Martin Boeree); University College of London, London, UK (Anna Bateson, Timothy McHugh, Kasha Singh, Robert Hunt, Alimuddin Zumla); MRC Clinical Trials Unit at UCL, London, UK (Andrew J Nunn, Patrick PJ Phillips, Sunita Rehal); University of Cape Town, Cape Town, South Africa (Rodney Dawson, Kim Narunsky); University of Stellenbosch, Cape Town, South Africa (Andreas Diacon, Jeannine du Bois, Amour Venter, Sven Friedrich); University of the Witswatersrand, Johannesburg, South Africa (Ian Sanne, Karla Mellet, Eefje de Jong); The Aurum Institute, Johannesburg, South Africa (Gavin Churchyard, Salome Charalambous, Nomagugu Ndlovu, Vinodh Edward, Madulagotla Sebe, Lungile Mbata, Robert Wallis); University of Zambia, Lusaka, Zambia (Peter Mwaba); NIMR-Mbeya Medical Research Centre, Mbeya, Tanzania (Nyanda Elias Ntinginya, Chacha Mangu, Christina Manyama, Gabriel Rojas-Ponce, Bariki Mtafya, Leonard Maboko); Ifakara Health Institute, Bagamoyo, Tanzania (Lilian T. Minja, Mohamed Sasamalo); Swiss Tropical and Public Health Institute, Basel, Switzerland, University of Basel, Basel, Switzerland (Klaus Reither, Levan Jugheli) ; Kilimanjaro Clinical Research Institute, Moshi, Tanzania (Noel Sam, Gibson Kibiki, Hadija Semvua, Stellah Mpagama); Medical Research Unit - Albert Schweitzer Hospital,

Lambarene, Gabon (Abraham Alabi, Ayola Akim Adegnika); Kenya Medical Research Institute, Nairobi, Kenya (Evans Amukoye); Makerere University, Kampala, Uganda (Alphonse Okwera).

Supplementary methods:

Data management

Clinical data were entered by study staff on electronic source tablet PCs with pre-programmed edit checks (Clinical Ink, Winston Salem, NC), with weekly electronic transfers to the core database. TB lab results were processed by double data entry or electronic transfer, depending on lab, and underwent 100% source data verification. ECG data and South African safety lab data were uploaded electronically; Tanzanian safety lab data were entered manually.

Definition of primary endpoint

Two consecutive negative sputum cultures without intervening positive, contaminated or missing cultures were required for the primary endpoint. Intermittent missing or contaminated culture results between follow up visits were effectively imputed as positive (i.e. assuming worst case scenario). All primary and secondary analyses were done on the modified intention-to-treat population (all randomized patients without evidence of rifampicin resistance on phenotypic test who took at least one dose of study treatment with an available outcome and who had at least one positive culture result at screening, week 1 or week 2 either on liquid or solid media).

Sensitivity analyses and analysis of secondary endpoints

The following secondary efficacy endpoints were pre-specified in the statistical analysis plan:

- Time to stable culture conversion to negative (defined as above) on solid culture up to 12 weeks

- Time to stable culture conversion to negative (defined as above) in liquid culture up to 26 weeks
- Time to stable culture conversion to negative (defined as above) on solid culture up to 26 weeks

The following sensitivity analyses on the primary endpoint were pre-specified in the statistical analysis plan:

- Time to stable culture conversion to negative in liquid culture up to 12 weeks ignoring missing and contaminated cultures, ie time to first of two consecutive negative cultures without intervening positive cultures, irrespective of missing and contaminated cultures.
- Time to stable culture conversion to negative on solid culture up to 12 weeks ignoring missing and contaminated cultures, ie time to first of two consecutive negative cultures without intervening positive cultures, irrespective of missing and contaminated cultures.

In post hoc analyses, time to stable culture conversion to negative in liquid culture and on solid culture up to 8 weeks was done for comparability with previous TB phase II trials and also the same endpoints ignoring missing and contaminated cultures as post hoc sensitivity analyses.

Two further post hoc analyses: a repeat of the primary analysis of the primary endpoint excluding patients with resistance to drug to which they were allocated, and an analysis of time to stable culture conversion to negative on solid culture up to 12 weeks excluding patients without evidence of culture positivity on solid media.

Pharmacokinetic sub-study

20 patients in each treatment arm, stratified from two Tanzanian and two South African sites, were enrolled for pharmacokinetics sampling. After an overnight fast, patients were hospitalized and took all TB drugs with a standardized light meal and serial venous blood

samples were taken at pre-dose and at 1, 2, 3, 4, 6, 8, 12, and 24h post dose. Separated plasma was frozen within one hour after collection of blood and transported on dry ice for bioanalysis in The Netherlands. Total (protein-unbound plus bound) plasma concentrations of RIF, H, Z, E and moxifloxacin were measured by validated Ultra Performance or High Performance Liquid Chromatography methods with ultraviolet or fluorescence detection. Noncompartmental pharmacokinetic analysis was performed with Winnonlin version 5.3 (Pharsight Corp., Mountain View, California US) to yield pharmacokinetic parameters, including the area under the plasma concentration versus time curve (AUC0-24h or total exposure) and highest observed plasma concentration (Cmax). Pharmacokinetic parameters were presented descriptively and were log-transformed before further statistical analysis. This statistical analysis included comparison of exposures between arms, in which AUC_{0-24h} and C_{max} values for rifampicin, isoniazid and pyrazinamide (used in all five study arms) were compared with a one-way analysis of variance (ANOVA) on logarithmically transformed AUC values, using Tukey HSD test as post hoc test. AUC_{0-24h} values for ethambutol, which was used in two arms, were compared with an independent samples T-test on logarithmically transformed AUC values. Statistical analysis also included an evaluation of the effect of patient characteristics (gender, weight, BMI, age, ethnicity) on the exposures achieved.

Supplementary Tables and Figures

Table S1: Inclusion and Exclusion criteria

Inclusion Criteria
Each patient must meet all of the following inclusion criteria prior to enrolment into the study:
1. The patient has given free, signed written or witnessed oral informed consent for study participation prior to all trial-related procedures, including HIV testing if HIV serostatus is not known or the last documented negative is more than four weeks ago.
2. The patient has a diagnosis of pulmonary tuberculosis from a health clinic established by sputum smear and/or GeneXpert MTB/RIF® and/or chest X-ray.
3. An adequate sputum bacterial load is confirmed by a Ziehl-Neelsen stained smear in the study laboratory, done from concentrated sputum found at least 1+ on the IUATLD/WHO scale.
4. The patient has rapid tests performed in the study laboratory from the screening sputum sample(s), with the following results:<br>- GeneXpert MTB/RIF®positive for MTB complex, and indicating susceptibility to Rifampicin,<br>- Line probe assay (e.g. HAIN MTBDR*plus*) result not indicating resistance to rifampicin or isoniazid.<br>- In case of an inconclusive result, this test is to be repeated at least once. Should the repeat test result again be inconclusive, the patient may continue with enrolment without this result.
5. The patient is aged at least 18 years at the day of informed consent.
6. The patient has a body weight in light clothing and without shoes of at least 35 kg, but not more than 90 kg.
7. Female patients of childbearing potential must have a negative serum pregnancy test, and consent to practise an effective method of birth control until week 26.<br>Effective birth control for female patients has to include two methods, including methods that the patient's sexual partner(s) use. At least one must be a barrier method.<br>Female patients are considered not to be of childbearing potential if they are post-menopausal with no menses for the last 12 months, or surgically sterile (this condition is fulfilled by bilateral oophorectomy, hysterectomy, and by tubal ligation which is done at least 12 months prior to enrolment).
8. Male patients must consent to use an effective contraceptive method, if their sexual partner(s) is/are of childbearing potential, and if they are not surgically sterile (see 6.).<br>Contraception by male participants must be practised until at least week 24 to cover the period of spermatogenesis. Contraceptive methods used by male participants may include hormonal methods used by the partner(s).
9. The patient has a firm home address that is readily accessible for visiting and willingness to inform the study team of any change of address during trial participation, or will be compliant to study schedule, in the discretion of the investigator.

Exclusion Criteria
Patients for whom one of the following criteria is met will be excluded from trial:
1. Circumstances that raise doubt about free, uncoerced consent to study participation (e.g. in a prisoner or mentally handicapped person)
2. Poor General Condition where delay in treatment cannot be tolerated or death within three months is likely.
3. The patient is pregnant or breast-feeding.
4. The patient has an HIV infection, and is receiving antiretroviral treatment (ART), and/or has less than 200 cd4 cells/µl, and/or is likely to require ART during the twelve weeks of experimental study treatment. Inclusion of HIV positive patients is only possible with specific approval from the site's local ethics committee.
5. The patient has a known intolerance to any of the study drugs, or concomitant disorders or conditions for which SQ109, rifampicin, moxifloxacin, or standard TB treatment are contraindicated.
6. The patient has an history or evidence of clinically relevant metabolic, gastrointestinal, neurological, psychiatric or endocrine diseases, malignancy, or any other condition that will influence treatment response, study adherence or survival in the judgement of the investigator, especially:<br>– clinically significant evidence of severe TB (e.g. miliary TB, TB meningitis. Limited lymph node involvement will not lead to exclusion)<br>– serious lung conditions other than TB or severe respiratory impairment in the discretion of the investigator;<br>– neuropathy, epilepsy or significant psychiatric disorder;<br>– uncontrolled and/or insulin-dependent diabetes<br>– cardiovascular disease such as myocardial infarction, heart failure, coronary heart disease, uncontrolled hypertension (systolic blood pressure ≥160 mmHg and/or diastolic blood pressure of ≥100 mmHg on two occasions), arrhythmia, or tachyarrhythmia.<br>– long QT syndrome (see criterion 9.), or family history of long QT syndrome or sudden death of unknown or cardiac-related cause<br>– *Plasmodium spp*. parasitemia as indicated by thick blood smear or a positive rapid test present at screening<br>– Alcohol or other drug abuse that is sufficient to significantly compromise the safety or cooperation of the patient, includes substances prohibited by the protocol, or has led to significant organ damage at the discretion of the investigator.
7. History of previous TB within the last five years.

8. Laboratory: at screening one or more of the following abnormalities were observed for the patient in screening laboratory:
   - Serum amino aspartate transferase (AST) and/or serum alanine aminotransferase (ALT) activity >3x the upper limit of normal
   - Serum total bilirubin level >2.5 times the upper limit of normal
   - Creatinine clearance (CrCl) level lower than 30 mls/min
   - Complete blood count with hemoglobin level <7.0 g/dL
   - Platelet count <50,000/mm3
   - Serum potassium below the lower level of normal

9. ECG findings in the screening ECG:
   - QTcF of >0.450 s
   - atrioventricular (AV) block with PR interval > 0.20 s,
   - prolongation of the QRS complex over 120 milliseconds,
   - other changes in the ECG that are clinically relevant as per discretion of the investigator.

10. The patient has had treatment with any other investigational drug within 1 month prior to enrolment, or enrolment into other clinical (intervention) trials is planned during week 1-26

11. Previous anti-TB treatment: the patient has had previous treatment with drugs active against M. tuberculosis within the last 3 months, including but not limited to INH, EMB, RIF, PZA, amikacin, cycloserine, rifabutin, streptomycin, kanamycin, para-aminosalicylic acid, rifapentine, thioacetazone, capreomycin, fluoroquinolones, thioamides.

12. QT prolonging medications: Administration within 30 days prior to study start, anticipated administration during the study period, or during the 12 weeks of experimental treatment, of any QT-prolonging agents such as, but not limited to, azithromycin, bepridil chloroquine, chlorpromazine, cisapride, cisapride, clarithromycin, disopyramide dofetilide, domperidone, droperidol, erythromycin, halofantrine, haloperidol, ibutilide, levomethadyl, lumefantrine, mefloquine, mesoridazine, methadone, moxifloxacin, pentamidine, pimozide, procainamide, quinidine, quinine, roxithromycin, sotalol, sparfloxacin, terfenadine, thioridazine.

    Exceptions may be made for participants who have received 3 days or less of one of these drugs or substances, if there has been a wash-out period equivalent to at least 5 half-lives of that drug or substance.

    Patients who have ever received amiodarone will be excluded from study participation.

13. CYP 450 inducers/inhibitors: administration within 30 days prior to dosing, or planned administration until the end of week 12, of any drug(s) or substance(s) known to be strong inhibitors or inducers of cytochrome P450 enzymes, or specific inhibitors/inducers of SQ109-metabolizing enzymes as listed in section 9.4.1 (p.56) and Appendix 3.

    Exceptions may be made for subjects that have received 3 days or less of one of these drugs or substances, if a wash-out period equivalent to at least 5 half-lives of that drug or substance prior to

study treatment is granted.

Table S2: Study drug and tablet scheme for intensive/experimental and continuation phase

	**Weight Band 1:**<br><br>**35-37 kgs**	**Weight Band 2:**<br><br>**38-54 kg**	**Weight Band 3:**<br><br>**55-70kg**	**Weight Band 4:**<br><br>**>70kg**
Control HRZE	RHZE: 2 tablets<br>Vit B6: 1 tablet	RHZE: 3 tablets<br>Vit B6: 1 tablet	RHZE: 4 tablets<br>Vit B6: 1 tablet	RHZE: 5 tablets<br>Vit B6: 1 tablet
Arm 1 (R_{35}): $HR_{35}ZE$	RHZE: 2 tablets<br>R300: 3 tablets<br>Vit B6: 1 tablet	RHZE: 3 tablets<br>R150: 1 tablet<br>R300: 3 tablets<br>Vit B6: 1 tablet	RHZE: 4 tablets<br>R300: 5 tablets<br>Vit B6: 1 tablet	RHZE: 5 tablets<br>R300: 7 tablets<br>Vit B6: 1 tablet
Arm 2 (Q): HRZQ	RHZ: 2 tablets<br>SQ109: 2 tablets<br>Vit B6: 1 tablet	RHZ: 3 tablets<br>SQ109: 2 tablets<br>Vit B6: 1 tablet	RHZ: 4 tablets<br>SQ109: 2 tablets<br>Vit B6: 1 tablet	RHZ: 5 tablets<br>SQ109: 2 tablets<br>Vit B6: 1 tablet
Arm 3 ($R_{20}Q$): $HR_{20}ZQ$	RHZ: 2 tablets<br>R150: 2 tablets<br>SQ109: 2 tablets<br>Vit B6: 1 tablet	RHZ: 3 tablets<br>R150: 3 tablets<br>SQ109: 2 tablets<br>Vit B6: 1 tablet	RHZ: 4 tablets<br>R150: 4 tablets<br>SQ109: 2 tablets<br>Vit B6: 1 tablet	RHZ: 5 tablets<br>R150: 5 tablets<br>SQ109: 2 tablets<br>Vit B6: 1 tablet
Arm 4 ($R_{20}M$) $HR_{20}ZM$	RHZ: 2 tablets<br>R150: 2 tablets<br>moxifloxacin: 1 tabl<br>Vit B6: 1 tablet	RHZ: 3 tablets<br>R150: 3 tablets<br>moxifloxacin: 1 tabl<br>Vit B6: 1 tablet	RHZ: 4 tablets<br>R150: 4 tablets<br>moxifloxacin: 1 tabl<br>Vit B6: 1 tablet	RHZ: 5 tablets<br>R150: 5 tablets<br>moxifloxacin: 1 tabl<br>Vit B6: 1 tablet
All treatment arms: continuation phase	RH: 2 tablets<br><br>Vit B6: 1 tablet	RH: 3 tablets<br><br>Vit B6: 1 tablet	RH: 4 tablets<br><br>Vit B6: 1 tablet	RH: 5 tablets<br><br>Vit B6: 1 tablet

RHZ=: 150 mg rifampicin, 75 mg isoniazid and 400 mg pyrazinamide;
RHZE = 150 mg rifampicin, 75 mg isoniazid, 400 mg pyrazinamide and 275 mg ethambutol
R150 = 150 mg rifampicin
R300 = 300 mg rifampicin

M = Moxifloxacin
Vit B6 = Pyridoxine 25mg
RH: 150 mg rifampicin, 75 mg isoniazid

Table S3. Summary of grade 3 or above adverse events by MedDRA system organ class.

Table shows the number of patients experiencing each event by grade. Where a patient experienced multiple events of the same system organ class, only the most severe is included in the table.

	Control		$RIF_{35}HZE$			RIFQHZ		$RIF_{20}QHZ$		$RIF_{20}MHZ$		Total		
**Severity Grading**	**3**	**4**	**3**	**4**	**5**	**3**	**4**	**3**	**4**	**3**	**4**	**3**	**4**	**5**
Investigations	5(4%)	0	1(2%)	0	0	1(2%)	0	4(7%)	0	2(3%)	1(2%)	**13(4%)**	**1(<0.5%)**	**0**
Metabolism and nutrition disorders	1(1%)	1(1%)	0	3(5%)	0	0	0	1(2%)	0	0	0	**2(1%)**	**4(1%)**	**0**
Hepatobiliary disorders	1(1%)	0	1(2%)	2(3%)	0	1(2%)	0	0	1(2%)	0	0	**3(1%)**	**3(1%)**	**0**
Blood and lymphatic system disorders	2(2%)	0	0	0	0	1(2%)	0	0	0	1(2%)	0	**4(1%)**	**0**	**0**
Respiratory, thoracic and mediastinal disorders	1(1%)	0	0	0	0	1(2%)	0	1(2%)	0	1(2%)	0	**4(1%)**	**0**	**0**
Gastrointestinal disorders	0	0	1(2%)	0	0	0	0	1(2%)	0	0	1(2%)	**2(1%)**	**1(<0.5%)**	**0**
General disorders and administration site conditions	0	0	1(2%)	0	1(2%)	0	0	0	0	1(2%)	0	**2(1%)**	**0**	**1(<0.5%)**
Infections and infestations	1(1%)	0	0	0	0	1(2%)	1(2%)	0	0	0	0	**2(1%)**	**1(<0.5%)**	**0**
Psychiatric disorders	0	1(1%)		0	0	0	1(2%)		0		1(2%)		**3(1%)**	**0**
Other*	2(2%)	0	1(2%)	0	0	1(2%)	0	2(4%)	0	2(3%)	0	**8(2%)**	**0**	**0**
**Overall**	**11(9%)**	**2(2%)**	**3(5%)**	**5(8%)**	**1(2%)**	**5(8%)**	**2(3%)**	**6(11%)**	**1(2%)**	**6(10%)**	**3(5%)**	**31(8%)**	**14(4%)**	**1(<0.5%)**

* Other grade 3 events include 3 musculoskeletal and connective tissue disorders (1 Q, 2 R20M);2 vascular disorders (1 control, 1 R20q); 1 eye disorder (R20Q); 1 renal and urinary disorder (R35); 1 injury, poisoning and procedural complication (Control);

Table S4. Pharmacokinetics of TB drugs recorded after 4 weeks of treatment (steady-state)

		Control (RIFHZE) (n=19)	$RIF_{35}HZE$ (n=20)	RIFQHZ (n=19)	$RIF_{20}QHZ$ (n=19)	$RIF_{20}MHZ$ (n=19)
Drug		Geometric mean (range)	Geometric mean (range)	Geometric mean (range)	Geometric mean (range)	Geometric mean (range)
Rifampicin	$AUC_{0\text{-}24h}$	24.2 (11.9-52.5)	170 (103-266)	17.4 (4.3-45.3)	68.3 (38.5-149)	57.8 (15.0-121)
	C_{max}	5.8 (2.1-12.0)	26.7 (15.0-39.1)	3.4 (0.7-8.9)	11.6 (6.6-23.8)	11.3 (2.3-25.5)
	T_{max} *	3.1 (1.0-4.1)	4.0 (2.0-6.0)	4.0 (1.0-6.1)	4.0 (2.0 -6.0)	3.1 (2.0-8.0)
	Cl/F	0.41 (0.19-0.84)	0.21 (0.13-0.34)	0.59 (0.22-2.3)	0.29 (0.13-0.52)	0.35 (0.17-1.3)
	Vd/F	1.0 (0.51-3.8)	0.75 (0.42-1.3)	1.6 (0.45-4.8)	0.87 (0.62-2.1)	0.91 (0.44-5.7)
	$t_{1/2}$	1.8 (1.3-3.1)	2.5 (1.6-4.0)	1.8 (1.3-3.5)	2.1 (1.2-4.0)	1.8 (1.3-2.9)
Isoniazid	$AUC_{0\text{-}24h}$	11.1 (4.4-32.2)	9.1 (3.8-30.8)	11.3 (2.5-25.8)	8.4 (2.9-19.7)	10.3 (2.6-23.1)
	C_{max}	2.4 (1.4-4.5)	2.0 (0.9-4.1)	1.9 (0.5-3.6)	1.6 (0.7-2.8)	1.8 (0.6-3.8)
	T_{max} *	3.0 (0.5-4.1)	3.0 (1.0-6.0)	3.9 (1.0-6.0)	3.2 (2.0-6.0)	3.3 (1.0-6.1)
	Cl/F	0.45 (0.16-1.1)	0.55 (0.16-1.3)	0.44 (0.19-2.0)	0.60 (0.25-1.7)	0.48 (0.22-1.9)

	Vd/F	2.1 (1.4-5.8)	2.6 (0.96-7.8)	2.4 (1.3-9.9)	2.7 (1.5-5.4)	2.6 (1.3-7.1)
	$t_{1/2}$	3.2 (1.9-6.6)	3.3 (1.9-7.0)	3.8 (1.9-7.9)	3.1 (1.4-5.3)	3.8 (1.5-6.5)
Pyrazinamide	AUC_{0-24h}	361 (255-514)	306 (212-567)	339 (232-646)	324 (241-631)	350 (216-580)
	C_{max}	33.5 (22.8-46.0)	31.3 (24.6-43.2)	27.8 (19.1-44.2)	27.3 (21.1-41.8)	30.1 (20.9-42.5)
	T_{max} *	3.0 (1.0-4.1)	3.0 (1.8-6.1)	4.0 (2.0 -8.0)	4.0 (2.0-6.1)	4.0 (2.0-8.0)
	Cl/F	0.070 (0.049-0.098)	0.082 (0.044-0.12)	0.074 (0.039-0.11)	0.077 (0.040-0.10)	0.071 (0.043-0.12)
	Vd/F	0.71 (0.57-0.99)	0.69 (0.47-0.91)	0.73 (0.54-0.92)	0.75 (0.63-0.97)	0.69 (0.52-0.89)
	$t_{1/2}$	7.1 (5.6-9.6)	5.8 (4.1-9.3)	6.8 (4.5-9.7)	6.7 (5.1-11.1)	6.7 (4.5-10.0)
Moxifloxacin	AUC_{0-24h}					23.5 (12.7-32.2)
	C_{max}					2.6 (1.4-4.0)
	T_{max} *					3.0 (0.6-6.1)
	Cl/F					17.0 (12.4-31.4)
	Vd/F					151 (101-247.0)
	$t_{1/2}$					6.1 (4.5-8.3)
Ethambutol	AUC_{0-24h}	19.1 (12.4-26.9)	17.9 (11.9-25.1)			
	C_{max}	2.4 (1.5-3.6)	2.2 (1.0-3.7)			

	T_{max} *	4.0 (1.0-6.0)	4.0 (1.8-8.0)			
	Cl/F	0.78 (0.56-1.2)	0.84<br>(0.60-1.3)			
	Vd/F	10.3 (7.8-17.2)	11.8<br>(4.6-32.9)			
	$t_{1/2}$	9.2 (6.6-12.3)	9.5<br>(3.1-26.3)			

$AUC_{0–24h}$ = area under the time versus concentration curve up to 24 h after the dose (in h*mg/L)
C_{max} = peak plasma concentration (in mg/L).
T_{max} = time to maximum concentration (in h)
Cl/F = clearance (in L/kg/h)
Vd/F = apparent volume of distribution (in L/kg)
F = bio-availability
$t_{1/2}$ = elimination half life (in h)

*: median (range)

Table S5. Summary of analyses of time to culture conversion under different definitions.

		Control	$RIF_{35}HZE$	RIFQHZ	$RIF_{20}QHZ$	$RIF_{20}MHZ$	Total
**Total in analysis (mITT)**		**123**	**63**	**58**	**56**	**63**	**363**
**Primary analysis to 12 weeks (MGIT culture) ignoring missing cultures**							
Adjusted hazard ratio (95%)*			1.58 (1.10, 2.27)	0.84 (0.57, 1.23)	0.79 (0.53, 1.17)	1.32 (0.92, 1.90)	
Stratified log-rank test			p = 0.018	p = 0.362	p = 0.123	p = 0.117	
Hazard ratio (95%), unadjusted			1.41 (0.99, 2.00)	0.87 (0.60, 1.28)	0.80 (0.54, 1.19)	1.26 (0.88, 1.80)	
Unstratified log-rank test			p = 0.043	p = 0.470	p = 0.247	p = 0.181	
**Primary analysis to 8 weeks (MGIT culture) ignoring missing cultures**							
Adjusted hazard ratio (95%)*			2.14 (1.32, 3.46)	1.15 (0.67, 1.96)	0.91 (0.50, 1.66)	1.97 (1.21, 3.20)	
Stratified log-rank test			p = 0.009	p = 0.934	p = 0.462	p = 0.014	
Hazard ratio (95%), unadjusted			1.78 (1.11, 2.85)	1.15 (0.68, 1.96)	0.82 (0.45, 1.49)	1.63 (1.02, 2.62)	
Unstratified log-rank test			p = 0.012	p = 0.590	p = 0.504	p = 0.033	
**Primary analysis to 12 weeks (LJ culture) ignoring missing cultures**							
Adjusted hazard ratio (95%)*			1.33 (0.97, 1.83)	0.90 (0.64, 1.25)	0.94 (0.68, 1.32)	0.80 (0.58, 1.11)	
Stratified log-rank test			p = 0.062	p = 0.892	p = 0.995	p = 0.369	
Hazard ratio (95%), unadjusted			1.36 (0.99, 1.87)	1.00 (0.72, 1.38)	1.01 (0.72, 1.40)	0.94 (0.69, 1.29)	
Unstratified log-rank test			p = 0.035	p = 0.989	p = 0.967	p = 0.698	
**Primary analysis to 8 weeks (LJ culture) ignoring missing cultures**							
Adjusted hazard ratio (95%)*			1.24 (0.88, 1.74)	0.97 (0.68, 1.38)	1.00 (0.70, 1.44)	0.78 (0.55, 1.10)	
Stratified log-rank test			p = 0.237	p = 0.930	p = 0.934	p = 0.254	
Hazard ratio (95%), unadjusted			1.23 (0.88, 1.72)	1.05 (0.74, 1.49)	1.06 (0.74, 1.51)	0.91 (0.65, 1.28)	
Unstratified log-rank test			p = 0.190	p = 0.743	p = 0.741	p = 0.568	

		Control	$RIF_{35}HZE$	RIFQHZ	$RIF_{20}QHZ$	$RIF_{20}MHZ$	Total
**Primary analysis to 12 weeks (MGIT culture) excluding patients with resistance to drugs to which they were allocated (phenotypic or genotypic tests).**							
**Total in Analysis**		**114**	**58**	**54**	**56**	**58**	**340**
**Adjusted hazard ratio (95%)***			1.91 (1.29, 2.82)	0.89 (0.58, 1.35)	0.81 (0.53, 1.25)	1.42 (0.96, 2.10)	
**Stratified log-rank test**			p = 0.001	p = 0.57	p = 0.34	p = 0.08	
**Hazard ratio (95%), unadjusted**			1.49 (1.02, 2.19)	0.95 (0.63, 1.43)	0.81 (0.53, 1.25)	1.42 (0.97, 2.08)	
**Unstratified log-rank test**			p = 0.04	p = 0.79	p = 0.35	p = 0.07	
**Time to first negative culture in liquid media to 12 weeks**							
**Total in Analysis**		**123**	**63**	**58**	**56**	**63**	**363**
**Adjusted hazard ratio (95%)***			1.59 (1.15, 2.20)	0.85 (0.60, 1.20)	0.83 (0.58, 1.17)	1.20 (0.87, 1.67)	
**Stratified log-rank test**			p = 0.010	p = 0.427	p = 0.160	p = 0.502	
**Hazard ratio (95%), unadjusted**			1.50 (1.08, 2.07)	0.94 (0.67, 1.31)	0.88 (0.62, 1.24)	1.17 (0.85, 1.62)	
**Unstratified log-rank test**			p = 0.008	p = 0.679	p = 0.405	p = 0.282	
**Time to first negative culture on solid media to 12 weeks**							
**Total in Analysis**		**123**	**63**	**58**	**56**	**63**	**363**
**Adjusted hazard ratio (95%)***			1.20 (0.88, 1.63)	1.29 (0.94, 1.77)	0.98 (0.71, 1.35)	0.77 (0.56, 1.05)	
**Stratified log-rank test**			p = 0.091	p = 0.002	p = 0.893	p = 0.107	
**Hazard ratio (95%), unadjusted**			1.22 (0.90, 1.67)	1.33 (0.97, 1.83)	1.05 (0.76, 1.44)	0.90 (0.64, 1.17)	
**Unstratified log-rank test**			p = 0.015	p = 0.048	p = 0.730	p = 0.237	

* Analysis adjusted for HIV status, GeneXpert cycle threshold (<16, ≥16), and site. MGIT analyses also adjusted for baseline TTP.

Table S6. Summary of primary analyses of time to culture conversion in ITT and PP populations.

	Control	$RIF_{35}HZE$	RIFQHZ	$RIF_{20}QHZ$	$RIF_{20}MHZ$	Total
**Total in analysis (ITT)**	**124**	**64**	**60**	**57**	**63**	**368**
**Primary analysis to 12 weeks (MGIT culture) in ITT population**						
**Adjusted hazard ratio (95%)***		1.78 (1.23 to 2.59)	0.83 (0.55 to 1.24)	0.78 (0.51 to 1.19)	1.41 (0.98 to 2.05)	
**Stratified log-rank test**		0.002	0.36	0.26	0.07	
**Hazard ratio (95%), unadjusted**		1.46 (1.02 to 2.11)	0.87 (0.59 to 1.30)	0.79 (0.52 to 1.19)	1.34 (0.93 to 1.93)	
**Unstratified log-rank test**		0.04	0.50	0.26	0.12	
**Total in analysis (PP)**	**122**	**62**	**57**	**56**	**62**	**359**
**Primary analysis to 12 weeks (MGIT culture) in Per Protocol population**						
**Adjusted hazard ratio (95%)***		1.82 (1.25 to 2.65)	0.87 (0.58 to 1.30)	0.76 (0.50 to 1.17)	1.39 (0.95 to 2.01)	
**Stratified log-rank test**		0.002	0.50	0.21	0.09	
**Hazard ratio (95%), unadjusted**		1.46 (1.01 to 2.10)	0.92 (0.62 to 1.37)	0.76 (0.50 to 1.16)	1.31 (0.91 to 1.90)	
**Unstratified log-rank test**		0.04	0.69	0.21	0.14	

* Analysis adjusted for HIV status, GeneXpert cycle threshold (<16, ≥16), and site. MGIT analyses also adjusted for baseline TTP.

Table S7 Proportion of patients converting to negative sputum culture in liquid and solid media at each time point during treatment.

	Weeks from randomisation	Control	$RIF_{35}HZE$	RIFQHZ	$RIF_{20}QHZ$	$RIF_{20}MHZ$
**Proportion converted on liquid media***	**4**	10%	15%	10%	13%	13%
	**6**	25%	44%	21%	22%	28%
	**8**	41%	58%	41%	31%	60%
	**12**	76%	81%	68%	68%	83%
	**17**	87%	86%	76%	90%	90%
	**26**	89%	88%	81%	92%	90%
**Proportion converted on solid media***	**4**	52%	63%	59%	63%	48%
	**6**	71%	77%	72%	73%	67%
	**8**	85%	94%	85%	88%	90%
	**12**	99%	100%	96%	96%	98%
	**17**	100%	100%	100%	100%	100%

* The Kaplan-Meier estimator has been used to estimate the proportions of patients converting to negative sputum culture by specific times as this more accurately accounts for censoring and loss to follow-up and corresponds to the data in Figure 2 in the main paper.

Figure S1: dose per body weight of all drugs dosed according to weight.

Plotted points correspond to study patients.

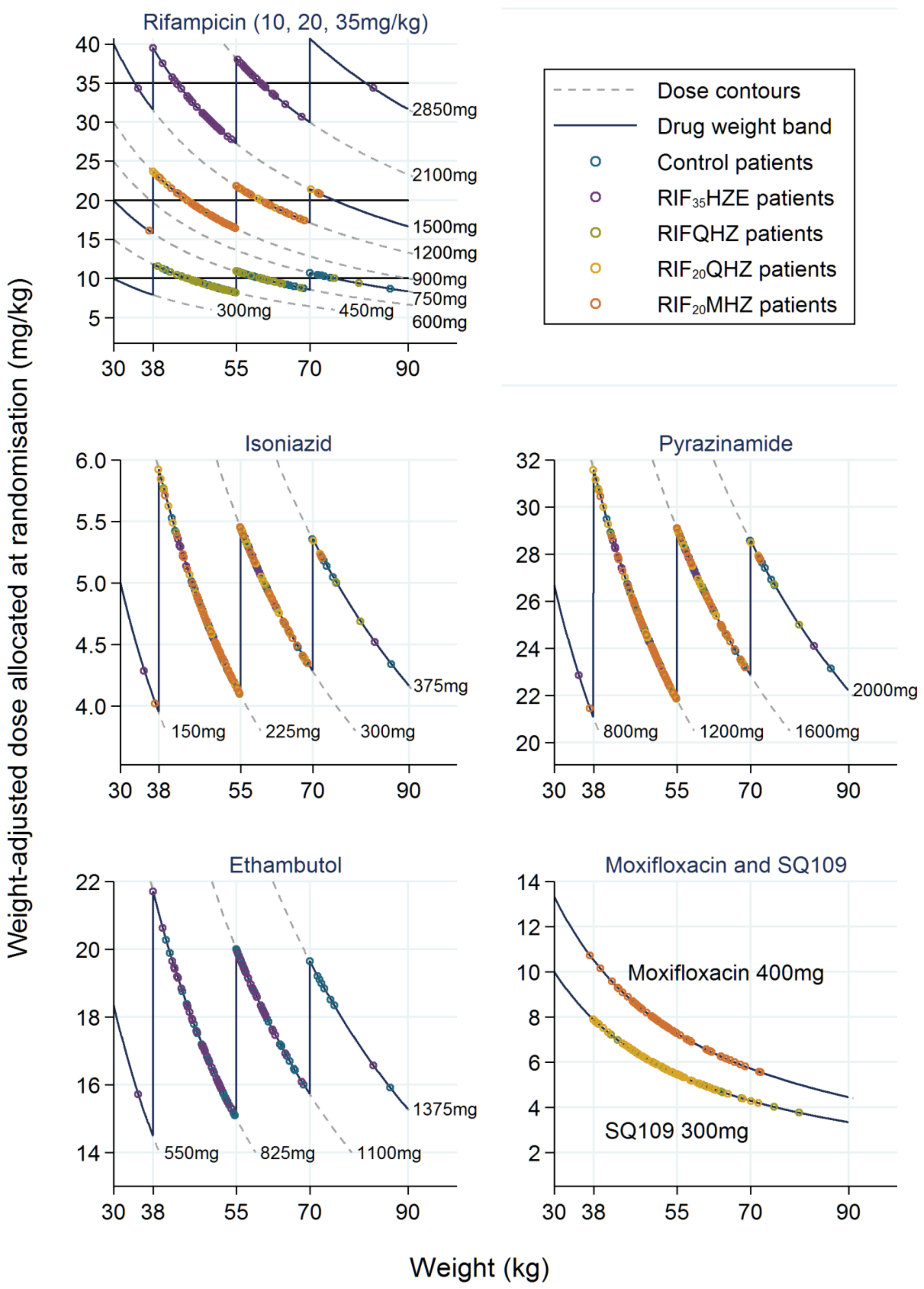

Figure S2. Pharmacokinetics of rifampicin

A. Pharmacokinetic curves of rifampicin (means and 1 standard deviation)

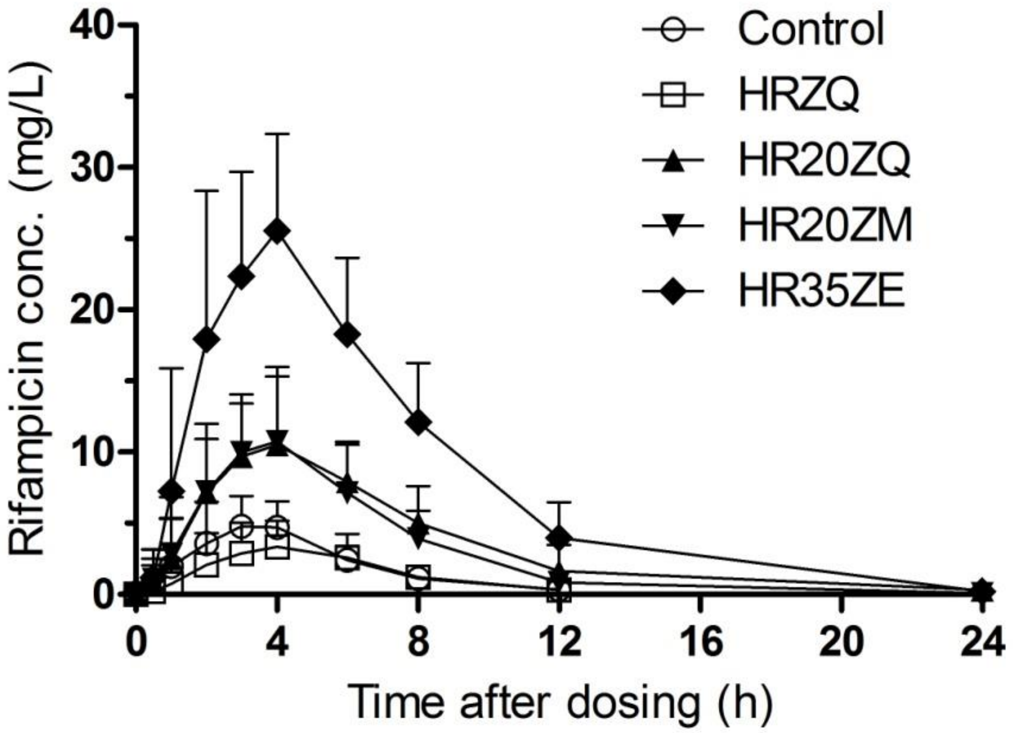

B. Distribution of total exposure to rifampicin ($AUC_{0–24h}$) by weight-adjusted dose and treatment arm. Horizontal lines indicate geometric mean values.

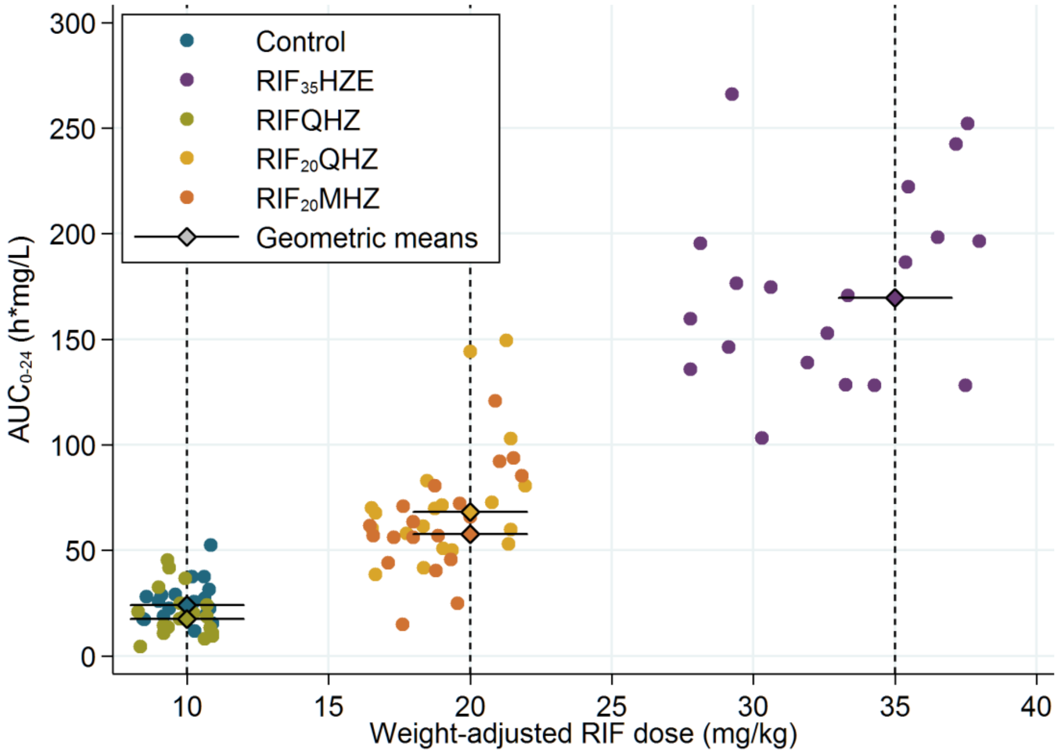

C. Distribution of peak plasma concentration of rifampicin (C_{max}) by weight-adjusted dose and treatment arm. Horizontal lines indicate geometric mean values.

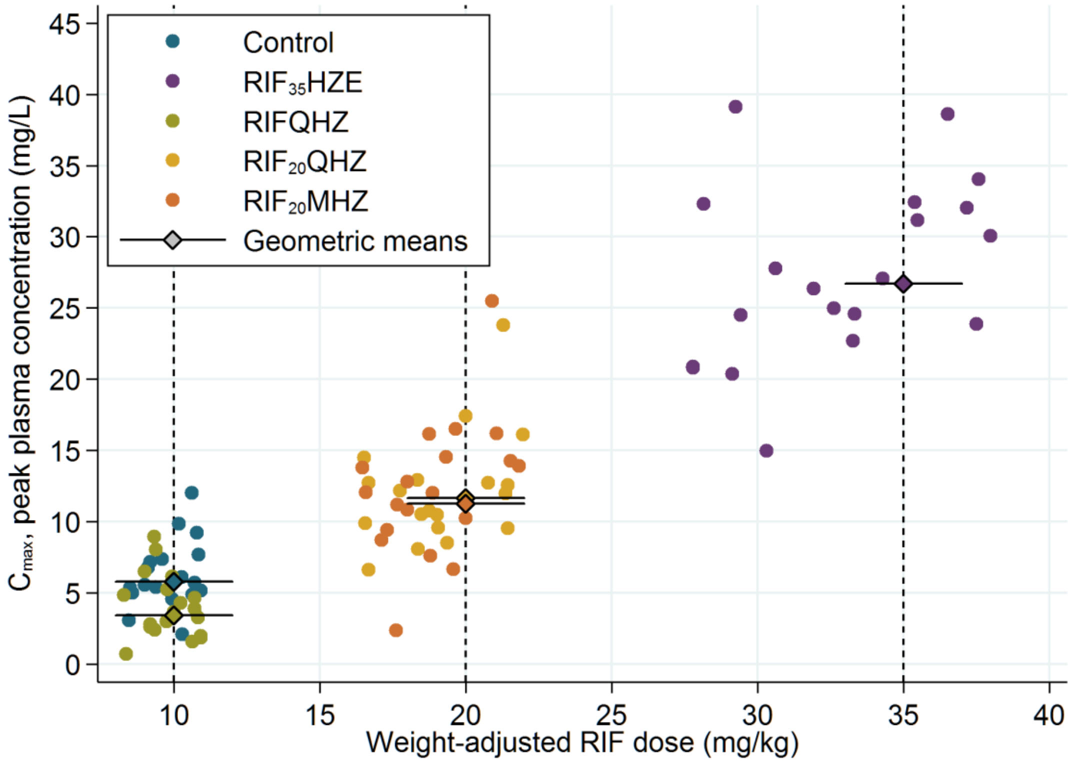

Supplement: Supplementary appendix [file mmc1.pdf]
